# CRX Expression in Pluripotent Stem Cell‐Derived Photoreceptors Marks a Transplantable Subpopulation of Early Cones

**DOI:** 10.1002/stem.2974

**Published:** 2019-01-30

**Authors:** Joseph Collin, Darin Zerti, Rachel Queen, Tiago Santos‐Ferreira, Roman Bauer, Jonathan Coxhead, Rafiqul Hussain, David Steel, Carla Mellough, Marius Ader, Evelyne Sernagor, Lyle Armstrong, Majlinda Lako

**Affiliations:** ^1^ Institute of Genetic Medicine Newcastle University Newcastle United Kingdom; ^2^ Genomics Core Facility Newcastle University, Newcastle United Kingdom; ^3^ CRTD/Center for Regenerative Therapies Dresden, Center for Molecular and Cellular Bioengineering Technische Universität Dresden Dresden Germany; ^4^ Institute of Neuroscience Newcastle University Newcastle United Kingdom

**Keywords:** Photoreceptors, Pluripotent stem cells, CRX, Single cell RNA‐seq, *Pde6brd1* mice, Subretinal transplantation

## Abstract

Death of photoreceptors is a common cause of age‐related and inherited retinal dystrophies, and thus their replenishment from renewable stem cell sources is a highly desirable therapeutic goal. Human pluripotent stem cells provide a useful cell source in view of their limitless self‐renewal capacity and potential to not only differentiate into cells of the retina but also self‐organize into tissue with structure akin to the human retina as part of three‐dimensional retinal organoids. Photoreceptor precursors have been isolated from differentiating human pluripotent stem cells through application of cell surface markers or fluorescent reporter approaches and shown to have a similar transcriptome to fetal photoreceptors. In this study, we investigated the transcriptional profile of CRX‐expressing photoreceptor precursors derived from human pluripotent stem cells and their engraftment capacity in an animal model of retinitis pigmentosa (*Pde6brd1*), which is characterized by rapid photoreceptor degeneration. Single cell RNA‐Seq analysis revealed the presence of a dominant cell cluster comprising 72% of the cells, which displayed the hallmarks of early cone photoreceptor expression. When transplanted subretinally into the *Pde6brd1* mice, the CRX^+^ cells settled next to the inner nuclear layer and made connections with the inner neurons of the host retina, and approximately one‐third of them expressed the pan cone marker, Arrestin 3, indicating further maturation upon integration into the host retina. Together, our data provide valuable molecular insights into the transcriptional profile of human pluripotent stem cells‐derived CRX^+^ photoreceptor precursors and indicate their usefulness as a source of transplantable cone photoreceptors. Stem Cells *2019;37:609–622*

**Significance Statement**

Diseases affecting the retina, the light‐sensitive extension of the central nervous system, account for approximately 26% of global blindness. Human pluripotent stem cells have been shown to differentiate into various retinal cell types, including photoreceptors, which can be enriched by cell surface or fluorescent molecule tagging approaches. Molecular heterogeneity of photoreceptor precursors derived from human pluripotent stem cells and their capacity to engraft into a fast degenerative model of retinitis pigmentosa have been investigated. Data show that photoreceptor precursors characterized by CRX expression are largely homogenous and committed to an early cone phenotype. Upon transplantation into degenerated retinae, these precursors settle into the appropriate layer, make connections with the host interneurons, and mature into cones. Future work is needed to assess at the functional level whether the transplanted cells are able to restore vision in degenerative models of retinal disease.

## Introduction

Blindness represents an increasing global problem which is closely correlated with old age. One in three people over 65 are at risk for developing sight loss and 90% of visually impaired people are over the age of 65 1. Overall, there are an estimated over 30 million blind and partially sighted people in Europe with an average of 1 in 30 individuals experiencing sight loss. The major causes of blindness are cataracts, glaucoma, and age‐related macular degeneration 2. The latter accounts for 50% of blind and partially sighted registration with an estimated prevalence of ∼600,000 significantly visually impaired people in the U.K. and over 8 million worldwide. Antioxidants, neuronal survival agents and vascular endothelial growth factor inhibitors have been shown to slow disease progression; however, to date there are no treatments to restore lost retinal cells and improve visual function; thus, there is a pressing need for research into the replacement and/or reactivation of dysfunctional photoreceptors and the adjoining retinal pigment epithelium (RPE) cells, both of which are essential for normal retinal function.

Human embryonic stem cells (hESC) and induced pluripotent stem cells (hiPSC) have an intrinsic capacity to differentiate into self‐organized laminated retinal organoids which contain all the key retinal cell types that form synaptic connections, respond to light and electrophysiological stimuli and engraft in animal models of retinal degeneration 3, 4, 5, 6, 7, 8, 9, 10. These retinal organoids have been shown to recapitulate human retinal development 8 and moreover to yield a population of cone photoreceptors which are able to both integrate and undergo material transfer in an environment‐dependent manner 11. A growing number of recent publications have also indicated the usefulness of patient specific retinal organoids for disease modeling and toxicology studies 12, 13, 14.

Despite this progress, there is a need to improve the efficiency and scalability of retinal organoid production, optimize the differentiation protocols, and characterize the retinal cell types generated within the organoids. For cell based therapies, it is important to generate and enrich defined populations of retinal cells of interest (e.g., photoreceptors, RPE cells) and assess their transplantation into the context of host retinal environment. Various approaches have been used to selectively enrich retinal cell types from these complex organoids including immunostaining with cell surface markers 15, 16 or flow activated cell sorting using reporter labeled cell lines 17, 18, which harbor fluorescent markers of key photoreceptor transcription factors such as cone rod homeobox (*CRX*) or neural retina leucine zipper (*NRL*) genes. Enrichment of photoreceptors is, however, not sufficient for ensuring successful engraftment into an adult retina. Work performed in animal models has shown that the maturity of stem cell‐derived photoreceptor precursors is critical for ensuring the directed engraftment of cells into the correct retinal layer 19, 20. Hence it is of key importance to enrich hESC‐ and hiPSC‐derived photoreceptor precursors from the self‐organized retinal organoids, study in detail their molecular profile and resemblance to endogenous photoreceptors emerging during human embryonic and fetal development, and identify the optimal stage of differentiation from which the engraftable photoreceptors can be obtained in sufficient numbers.

Advances made in single cell transcriptomics have enabled studies of human fetal and hiPSC‐derived cone photoreceptors and have highlighted the expression of a large number of genes, which have not been previously associated with this cell type and are likely to reflect the developmental maturity of developing retinal cell types 21. Single cell transcriptomic approaches have also been used to investigate the multiple retinal cell lineages emerging with the hESC‐derived retinal organoids 22. However, identification of cell types is reliant on a limited set of marker genes often derived from gene expression analysis done from bulk cultures or single cell studies of adult mouse retina 23 and which may not necessarily reflect the transcriptomic profile of developing photoreceptors within the hESC‐ and hiPSC‐derived retinal organoids. In 2016, our group reported the successful generation of a reporter‐labeled hESC line, in which expression of the green fluorescent protein (GFP) was controlled by CRX, a key transcription factor in retinal development with predominant expression in postmitotic precursors 17. We have differentiated this cell line to retinal organoids and have enriched the postmitotic CRX expressing photoreceptor precursors by fluorescence activated cell sorting. We have performed single cell RNA‐seq of the CRX^+^ precursors to assess intercellular heterogeneity and in parallel we have tested their engraftment into an animal model of early onset severe retinal degeneration (*Pde6brd1*). Our data suggest that the 72% of CRX^+^ precursors are characterized by the expression of early cone markers, with a small minority (28%) expressing genes involved in cholesterol and mitochondrial biogenesis. Furthermore, we show that the CRX^+^ photoreceptor precursors integrate into the correct retinal layer and make synaptic connections with the host bipolar cells of *Pde6brd1* mice.

## Materials and Methods

### Human Pluripotent Stem Cell Culture and Differentiation

The hESC line harboring the GFP reporter at the 3′UTR of *CRX* locus was expanded in mTeSR1 (Stem Cell Technologies, Vancouver, BC, Canada) at 37°C and 5% CO_2_ on 6‐well plates precoated with Low Growth Factor Matrigel (Corning Life Sciences, Acton, MA). Differentiation to retinal organoids was performed as described in Mellough et al. 5 with minor modifications which included addition of 10 μM Y27632 dihydrochloride for the first 48 hours of differentiation and 10% fetal calf serum, T3 (40 ng/ml), taurine (0.1 mM), and retinoic acid (0.5 μM) from day 18 of differentiation. Retinal organoids were collected on day 90 for single cell RNA‐Seq studies and day 90 and 120 for subretinal transplants.

### Single Cell RNA‐Seq

#### *Generation of Single Cell cDNA Library for mRNA Sequencing*

Retinal organoids at day 90 of differentiation were dissociated to single cells using the Embryoid Body Dissociation Kit (Miltenyi Biotec, Bergisch Gladbach, Germany) following the manufacturer's instructions. CRX‐GFP^+^ cells were sorted using a BD FACS Aria IIu fluorescence‐activated cell sorter (BD Biosciences, San Diego, CA). Single cells were loaded onto the C1 single‐cell mRNA‐Seq IFC (10–17 μm; Fluidigm, San Francisco, CA). Cell capture efficiency was assessed using the Zeiss Axiovert imaging system and empty sites, or sites with more than one cell were excluded from further analysis. Array Control RNA Spikes (Thermo Fisher Scientific, Waltham, MA) were prepared and added to lysis mix as described in the Fluidigm protocol. Cell lysis, reverse transcription and cDNA amplification were performed using the SMART‐Seq v4 Ultra Low Input RNA Kit for the Fluidigm C1 System (Clontech, Palo Alto, CA). Full length cDNA libraries were prepared using the Illumina Nextera XT DNA library preparation kit (Illumina, San Diego, CA). Libraries were pooled and sequenced (2 × 75 bp) on the Ilumina NextSeq 500 using a Mid Output v2 kit. The remainder of the cells were processed for bulk RNA‐Seq or subretinal transplants.

#### *Read Alignment and Quantification*

FASTQ files were trimmed with Trimmomatic version 0.33 with the parameters: trailing = 20, minlength = 60, and end = “PE.” The human reference genome GRCh38.p7 version 25 from GenCode was concatenated with the Ambion spike sequences provided by Fluidigm to create a reference genome. “Comprehensive gene annotation” was used for annotation and STAR 2.4.0 was used for alignment. A STAR index was created using the reference genome and annotations used with read length set to 75. STAR default parameters were used for alignment. The SAM files produced by STAR were converted into BAM files using SAMtools1.3. Reads were quantified using HTSEQ 0.6.1 with these parameters ‐f bam, ‐r name, ‐a 4, ‐i gene_id, and ‐m union.

#### *Quality Control: Filtering Cells and Genes*

The Scater R package was used for quality control and initial visualization of the raw data. Cells that had fewer than 150,000 reads or 2,000 genes were removed from downstream analysis (Supporting Information Fig. S1A, S1B). Next, a list of housekeeping genes was obtained from the Scone R package. The majority of cells had count numbers of between 20,000 and 80,000 for these genes. Cells with count numbers below 20,000 were filtered from analysis (Supporting Information Fig. S1C). High levels of mitochondrial genes have been shown to be an indication of dead or poor quality cells, thus cells with higher than 10% of mitochondrial genes were filtered (Supporting Information Fig. S1D). Cells containing higher than 25% of Ambion spikes were also removed from the analysis (Supporting Information Fig. S1E). Genes were filtered from analysis if they were detected in fewer than two cells (after cell filtering). The data was normalized using SCRAN (Supporting Information Fig. S1F). After filtering stage, 69 cells and 14,887 genes passed quality control. Data was deposited in Gene Expression Omnibus under the accession number GSE112507.

#### *Clustering Analysis*

The cells were clustered using the SC3 R package. SC3 uses *k‐*means clustering to test multiple clustering solutions. Clustering solutions between 2 and 10 were evaluated. Silhouette analysis was used to assess the distance between the cells assigned to the resulting clusters. The majority of cells had a silhouette score of above 0.85 when *k* was equal to 2. Cells with silhouette scores below 0.85 were removed and the clustering analysis was repeated. A total of 59 cells passed this filtration step and were included for further analysis.

#### *Validation of Single Cell RNA‐Seq Method*

To validate the single cell technology, we compared gene expression measured in single cells with gene expression measured in cell populations from the same fluorescence‐activated cell sorting experiment. The bulk RNA‐seq data was normalized using the “DESeq2” package, which was also used for conducting the differential gene expression analysis between the CRX‐GFP^+^ and CRX‐GFP^−^ subpopulations. Benjamini–Hochberg procedure was applied to results to correct for multiple testing. Significantly differentially expressed genes were selected using a cut‐off of absolute logfold differences of 1.5 and adjusted *p*‐value of .05. The single cell count matrix was then summed by gene to obtain pooled expression measurements. The counts for the significantly differently expressed genes from the bulk RNA‐Seq counts and the pooled single counts were combined and the data were normalized using the estimate size factors function within DESEQ2. The correlation in gene expression between pooled single cells and the bulk RNA CRX^+^ and CRX^−^ samples was assessed using a Pearson correlation test.

### Subretinal Transplants

#### *Ethics Statement*

All experimental work performed in this study was in accordance with the United Kingdom Animals (Scientific Procedures) Act 1986 and carried out in accordance with protocols approved by the Animal Welfare and Ethics Committee of the Newcastle University. All efforts were made to minimize the number and the suffering of animals used in these experiments.

#### *Animals*

*C3H/HeNHsd‐Pde6brd1* mice (Charles River Laboratories, Wilmington, MA) were housed in the animal facility at the Institute of Genetic Medicine at University of Newcastle on a standard 12‐hour light/dark cycle at the same light levels throughout the experimental period. Animals were kept in ventilated cages with food and water ad libitum.

#### *Immune Suppression*

To prevent immune‐rejection of the transplanted human cells, daily subcutaneous injection of cyclosporine A (50 mg/kg per day) was administered to the recipient animals starting at 1 day before the transplantation and maintained throughout the experiment.

#### *Surgery and Transplantation*

Male and female mice were anesthetized with a single intraperitoneal injection of a mixture of ketamine (0.075 ml/100 g) and medetomidine (0.100 ml/100 g) in sterile water. Pupils were dilated using 1% tropicamide (Bausch & Lomb U.K. Limited, Surrey, U.K.); a topical anesthetic, oxybuprocaine hydrochloride 0.4% (Bausch & Lomb U.K. Limited, Surrey, U.K.) was also applied. Eyes were protected with 0.2% Carbomer 980 eye gel (Gel tears, Bausch & Lomb U.K. Limited, Surrey, U.K.) and a glass coverslip was placed over the cornea. Surgery was performed under a direct visual control using an operating microscope (Leica M‐651). After creation of a 33 g limbal incision, a sterile blunt 34 gauge needle, attached to a 5 μl Hamilton syringe, was passed transvitreally and positioned into the subretinal space. An 1 μl cell suspension was then slowly injected into the subretinal space, between the neural retina and the RPE in the superior retina recipients. The needle was left in place for 20 seconds to allow for re‐equilibration of intraocular pressure before slowly withdrawing. Both eyes were injected and Chloramphenicol ointment was applied postoperatively. Anesthesia was reversed using intraperitoneal injections of 0.1 mg/ml of Antisedan. Mice were placed on heat mats and received softened food until fully recovered.

#### *Histology and Immunohistochemistry*

Retinal organoids were fixed in 4% paraformaldehyde for 20 minutes, followed by three washes in phosphate‐buffered saline (PBS), incubated overnight in 30% sucrose in PBS, embedded in Optical Cutting medium (OCT; Cellpath, Powys, U.K.) and frozen at −20°C. Ten micrometers cryostat sections were collected onto Superfrost Plus slides and stored at −20°C in slide boxes prior to immunostaining. Transplanted mice were sacrificed 3 weeks after transplantation, eyes were enucleated and fixed in 4% paraformaldehyde for 1 hour at 4°C, incubated overnight in 30% sucrose solution, and embedded in OCT. Cryosections were cut at 20 μm thick and all sections were collected onto Superfrost Plus slides, air‐dried for 20 minutes at room temperature (RT), hydrated with PBS for 30 minutes and incubated with blocking solution containing 10% goat serum and 0.3% Triton X‐100 for 1 hour at RT. Slides were incubated with the appropriate primary antibody overnight 4°C (Supporting Information Table S1). After rinsing with PBS, sections were incubated with the secondary antibody for 2 hours at RT, rinsed and counter‐stained with Hoechst 33342 (Life Technologies, Rockville, MD). Alexa Fluor 488, 546, and 647 secondary antibodies (Thermo Fisher Scientific, Waltham, MA) were used at a 1:1,000 dilution. Negative controls were carried out by omitting the primary antibody. For the nuclear staining on the whole mount retinas, the samples were washed in PBS then, the counter‐stained Hoechst were incubated for 2 hours at 4° with shaking.

#### *Image Acquisition and Processing*

Retinal sections were viewed on a Zeiss Axio ImagerZ2 equipped with Apotome 2 and Zen 2012 blue software (Carl Zeiss, Jena, Germany). Objectives lens used were EC Plan Neofluar ×20/0.5 Ph2, EC Plan Neofluar ×40/1.3 Ph2, EC Plan Apochromat ×63/1.4 Ph3. Series of XZ optical sections (<1 μm thick) were taken at 1.0 μm steps throughout the depth of the section. Final images are presented as a maximum projection and adjusted for brightness and contrast in Adobe Photoshop CS6 (Adobe, San Jose, CA).

## Results

### Single Cell RNA‐Seq Reveals that CRX Expressing Cells Comprise a Dominant Population of Early Cone Photoreceptors During hESC Differentiation

**Figure 1:**
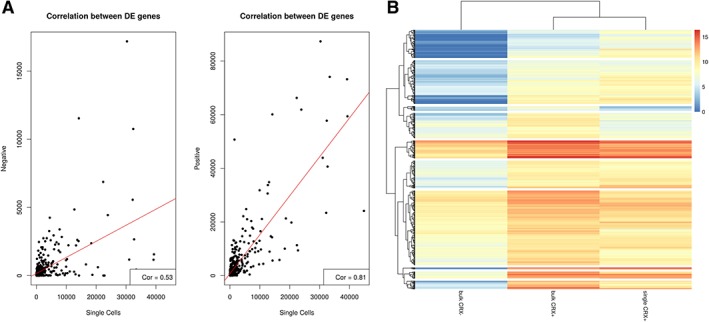
Correlation between bulk and single cell RNA‐seq analyses. **(A):** Correlation between bulk RNA‐Seq of CRX^−^ (left panel) and CRX^+^ (right panel) and single cell RNA‐Seq of CRX^+^ using the Pearson correlation test. **(B):** Comparative heatmap showing differentially expressed genes between bulk RNA‐Seq of CRX^+^ and CRX^−^ cells and single cell RNA‐seq of CRX^+^ cells.

In 2016, our group reported the derivation of a CRX‐GFP reporter hESC line and localization of CRX‐GFP expression in photoreceptor precursors, but not in the RPE, retinal ganglion cells (RGCs), or neurons of the developing inner nuclear layer (INL) following differentiation for 90 days 17. To investigate the transcriptional profile of these hESC‐derived‐photoreceptor precursors, we purified CRX^+^ cells at day 90 of retinal organoid differentiation using fluorescence‐activated cell sorting as reported in our previous publication 17 and performed bulk and single cell RNA‐Seq. After multiple steps of filtering and normalization (see Materials and Methods), 59 cells (from a total of 96) and 14,887 genes passed the quality control and were used for the remainder of the analysis. To validate the single cell analysis, we compared the gene expression measured by bulk RNA‐Seq data of CRX^+^ and CRX^−^ cells to a synthetic ensemble of single cell RNA‐Seq data of CRX^+^ obtained from the same fluorescence‐activated cell sorting experiment. This analysis revealed a high correlation in gene expression between single and bulk RNA‐Seq of CRX^+^ (*r* = .81) but not to bulk RNA‐Seq data of CRX^−^ cells (*r* = .53, Fig. 1A, 1B), thus indicating that the ensemble single cell RNA‐Seq data of CRX^+^ cells represents the transcriptional profile present in the bulk RNA‐Seq dataset of CRX^+^ cells.

**Figure 2:**
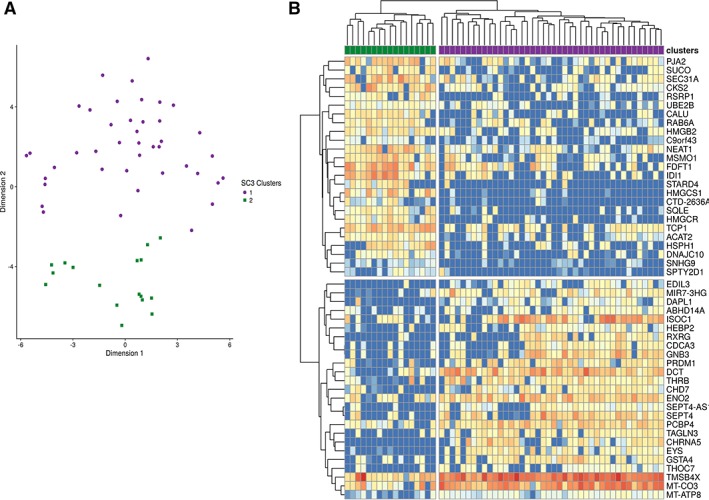
SC3 clustering analysis reveals two clusters within the CRX^+^ cells. **(A):** A predominant cluster containing 72% of cells (purple) and a smaller cluster containing 28% of cells (green) revealed by single cell RNA‐Seq of CRX^+^ cells. **(B):** Heatmap showing the 25 most differentially expressed genes between the two clusters within the CRX^+^ population.

**Figure 3:**
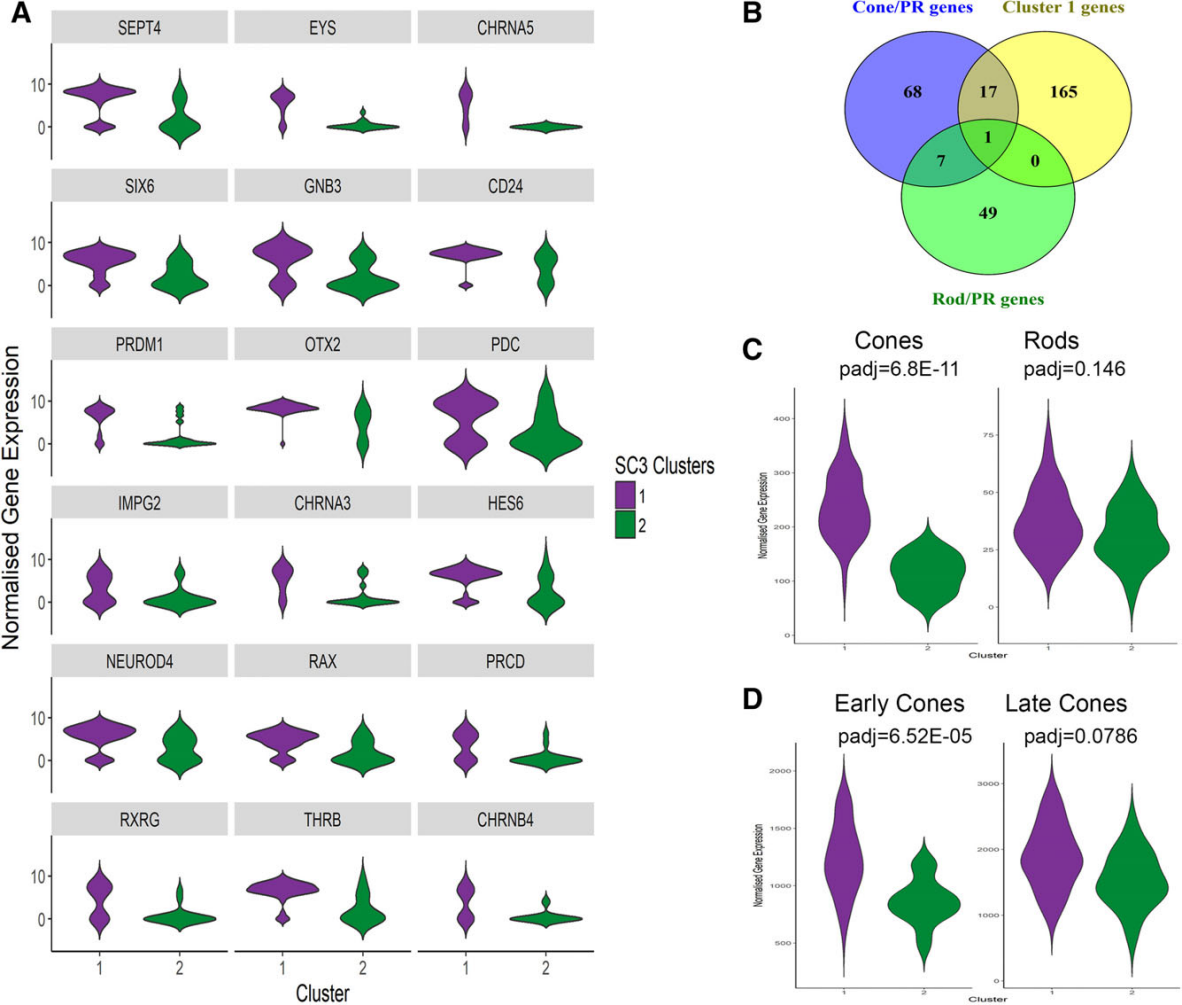
CRX^+^ cells within cluster 1 show an early cone transcriptional profile. **(A):** Significantly higher expression of photoreceptor and cone markers in cluster 1 shown through violin plot profiles of individual genes. **(B):** Venn diagram showing the overlap between cluster 1 genes and cone/photoreceptor gene set defined by Phillips et al. 22, but not rod/photoreceptor gene set. **(C):** The expression of cone and rod gene sets defined by Phillips et al. 22 was assessed in CRX^+^ clusters 1 and 2 through violin plots, showing a cone like expression profile for cluster 1 cells. **(D):** The expression of early and late cone gene sets defined by Welby et al. 21 was assessed in CRX^+^ clusters 1 and 2 through violin plots, showing an early cone like expression profile for cluster 1 cells. Abbreviation: PR, photoreceptor.

To investigate the transcriptional heterogeneity of the single CRX^+^ cells, we used an unsupervised clustering approach, SC3 24, which combines different clustering outcomes into a consensus matrix and displays each cell and differentially expressed genes within clusters. *K*‐means of 2 to 10 clusters were tested and 2 clusters were identified as optimal (Fig. 2A). Three hundred and twelve genes were significantly and differentially expressed between the two clusters (Supporting Information Table S2), and 25 of the most upregulated changed genes for each cluster are shown in Figure 2B. Cluster 1, the largest of two clusters (containing 72% of cells), was characterized by significantly higher expression of photoreceptor (*SEPT4, EYS, CHRNA5, SIX6, GNB3, CD24, PRDM1, OTX2, PDC, IMPG2, CHRNA3, HES6, NEUROD4, RAX, PRCD, ENO2, VTN, DCT,* and *DST*) and cone markers (*RXRY, THRB, CHRNB4, ISOC1,* and *PCBP4*; Fig. 3A). The differentially expressed genes from this cluster were compared with the cone/photoreceptor and rod/photoreceptor markers recently identified by Phillips et al. in pluripotent stem cell‐derived retinal organoids at day 218 of differentiation on the basis of coexpression with *CRX/PRDM1/THRB/RXRY* and *NR2E3/NRL*, respectively 22. While there was very little overlap between genes upregulated in cluster 1 with photoreceptor/rod markers (Fig. 3B), there was a significant overlap with photoreceptor/cone markers, albeit the difference in time point between the two different analyses (cluster 1 generated at day 90 of differentiation and photoreceptor/cone markers at day 218 of differentiation). In addition, there was hardly any overlap with the markers of RPE, RGCs, or retinal progenitor cells (RPCs) defined by Phillips et al. (data not shown). Next, we assessed the expression of cone and rod markers identified by Phillips et al. which indicated a significant increase in expression of cone markers in cluster 1 compared with cluster 2 (Fig. 3C); however, no significant changes were observed in the expression of rod markers between these 2 clusters (Fig. 3C). These data were corroborated by gene expression analysis, which showed no significant change in *NRL* expression between clusters 1 and 2 (Supporting Information Fig. S2A). Our immunohistochemical analysis indicated a low percentage of cells that coexpressed CRX and NRL (8.4%) in the retinal organoids at day 90 of differentiation (Supporting Information Fig. S2B, S2B′). Although this cannot be associated with cells residing in cluster 1 or 2, it probably reflects transitioning of rods through a cone‐like state as demonstrated in the developing murine retina 25.

We expanded our analysis of clusters 1 and 2 by further assessing the expression of early and late cone photoreceptor markers defined by Welby et al. 21. This analysis indicated a significantly higher expression of early cone markers in cluster 1 when compared with cluster 2 (Fig. 3D). Otx2 and Onecut1 factors have been shown to be coexpressed in Olig2+ retinal progenitor cells during early murine embryonic retinal development when cones and horizontal cells emerge; this coexpression is resolved as soon as the cones and horizontal cells are generated such that cones express Otx2, but not Onecut1 factors, and horizontal cells express Onecut1 factors but not Otx2 26. In accordance with this, we found that cluster 1 expressed significantly higher levels of *OTX2*, but not *ONECUT1* or *OLIG*2 (Supporting Information Fig. S3A). This was further corroborated by our immunostaining which revealed a significant overlap between the CRX‐GFP expression and OTX2 in day 90 retinal organoids (Supporting Information Fig. S3B); however, very few cells coexpressed CRX and OLIG2 or CRX and ONECUT1. Together, these data suggest that cluster 1 contains photoreceptors which have already committed to an early cone‐like phenotype.

**Figure 4:**
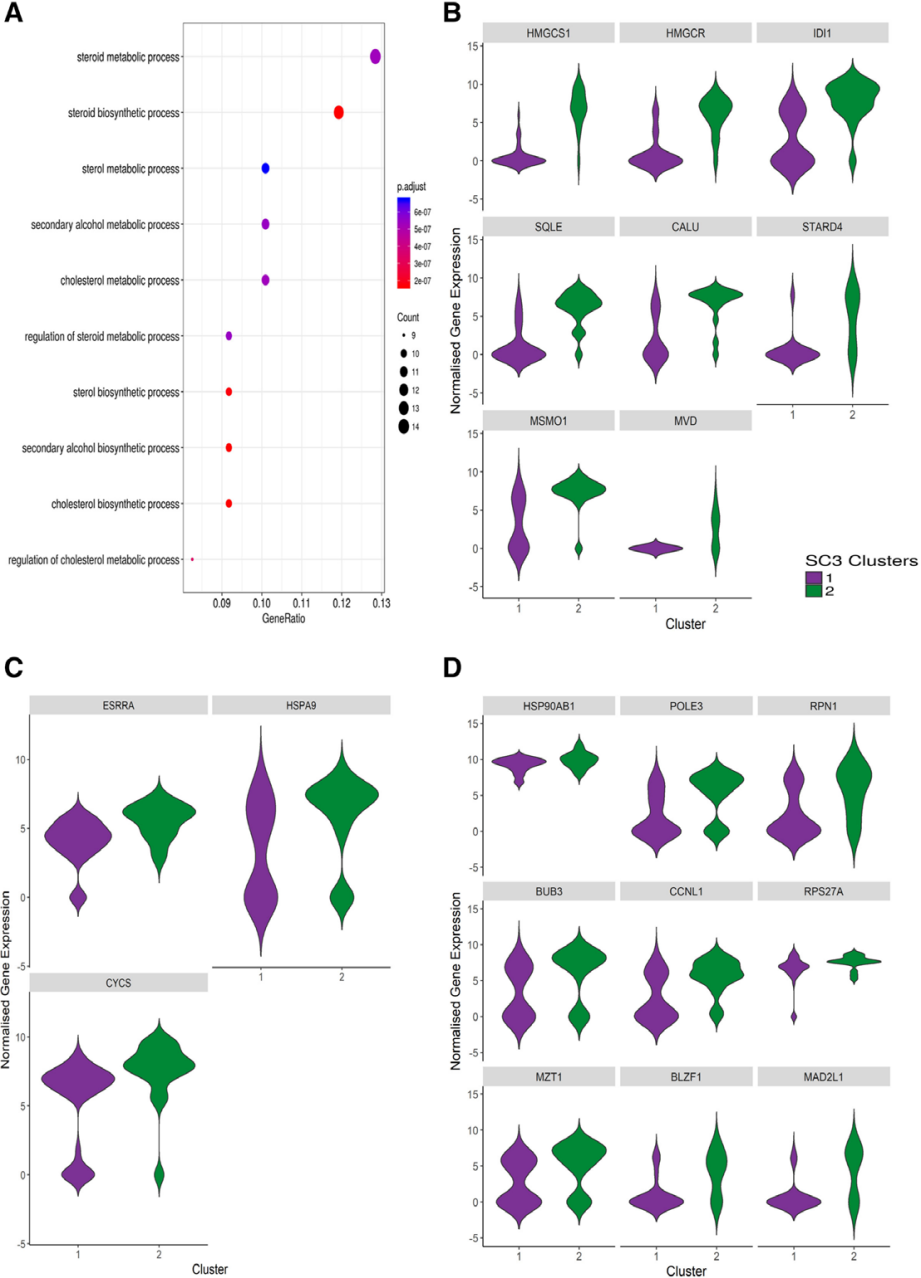
Cluster 2 shows enrichment of genes involved in cholesterol/lipid metabolism, mitochondrial biogenesis and mitotic genes. **(A):** Pathway analysis of genes enriched in cluster 2 showing the cholesterol and lipid metabolism to be the predominant pathways. **(B):** Violin plot analysis showing higher expression of genes involved in cholesterol/lipid metabolism genes in cluster 2 compared with cluster 1 CRX^+^ cells. **(C):** Violin plot analysis showing higher expression of genes involved in mitochondrial biogenesis in cluster 2 compared with cluster 1 CRX^+^ cells. **(D):** Violin plot analysis showing higher expression of genes involved in mitosis genes in cluster 2 compared with cluster 1 CRX^+^ cells.

Expression of other retinal markers expressed in interneurons and Müller glia cells during human retinal development was assessed across clusters 1 and 2; however, no enrichment or particular association of these lineage markers with cluster 2 were found (data not shown). It is of interest to note that the expression of *CRX* in cluster 2 was lower than cluster 1 (mean expression of 5.411 vs. 3.22), although these differences did not reach significance. Thus to gain more insights into cells represented in cluster 2, we first performed enrichment pathway analysis for genes that were significantly upregulated within this cluster (Supporting Information Table S2). This indicated that the cholesterol, sterol, and steroid biosynthetic processes were the most upregulated pathways within cluster 2 (Fig. 4A). The most highly upregulated genes in this cluster (Fig. 4B) comprised 3‐Hydroxy‐3‐Methylglutaryl‐CoA Synthase 1 (*HMGCS1*), 3‐Hydroxy‐3‐Methylglutaryl‐CoA Reductase (*HMGCR*), Isopentenyl‐Diphosphate Delta Isomerase 1 (*ID1*), Squalene Epoxidase (*SQLE*), StAR Related Lipid Transfer Domain Containing 4 (*STARD4*), Methylsterol Monooxygenase (*MSMO1*), and Farnesyl‐Diphosphate Farnesyltransferase 1 (*FDFT1*), Diphosphomevalonate decarboxylase (*MVD*), all of which are involved in various steps of cholesterol biosynthesis. Other genes involved in lipid synthesis such as Oxysterol‐binding protein 1 (*OSBP*), Dolichol‐phosphate mannosyltransferase (*DPM1*), Acyl‐CoA desaturase (*SCD*), Acetyl‐CoA acetyltransferase (*ACAT2*), Farnesyl pyrophosphate synthase (*FDPS*), ADP‐ribosylation factor (*ARF1*), golgi resident protein GCP60 (*ACBD3*), and low‐density lipoprotein receptor (*LDLR*) were also highly upregulated in cluster 2 when compared with cluster 1 (Supporting Information Table S2), thus suggesting that cholesterol/lipid biosynthesis is a key feature of cluster 2. In addition, a significant upregulation of genes involved in mitochondrial biogenesis and/or cristae formation (*ESRRA*, *HSPA9*, and *CYCS*; Fig. 4C) as well as genes involved in cell cycle/mitosis regulation (*HSP90AB1, POLE3, RPN1, BUB3, CCNL1, RPS27A, MZT1, BLZF1,* and *MAD2L1*; Fig. 4D) was observed in cluster 2.

**Figure 5:**
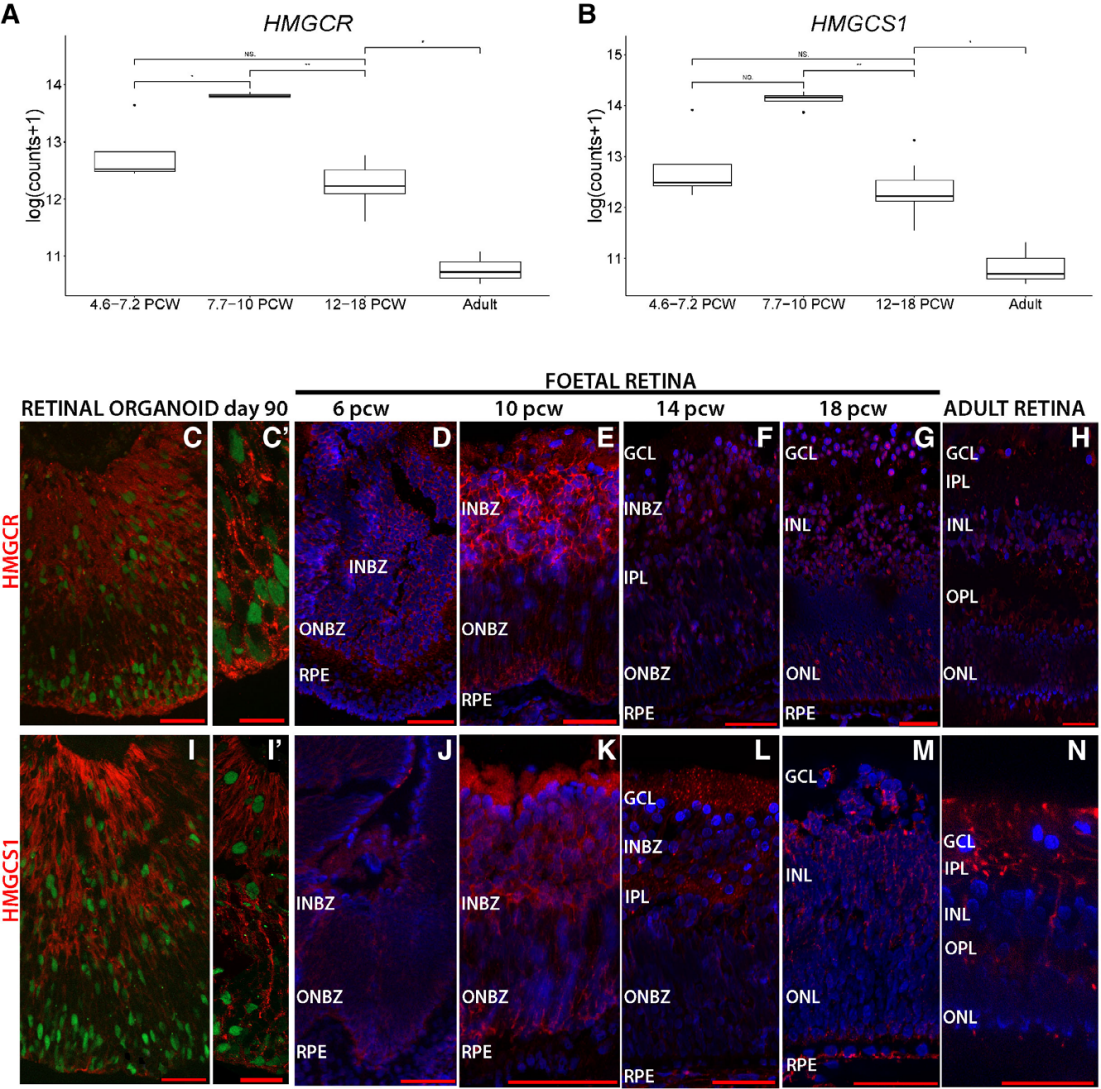
Expression HMGCR and HMGCS1 in retinal organoids, fetal, and adult human retina. **(A, B):** Box‐plots were obtained by grouping the samples into three developmental windows: 4.6–7.2 PCW, 7.7–10 PCW, 12–18 PCW, and samples from adult retina. Individual boxes quantify the distribution of the expression of selected genes in these associated windows. To this end, the logarithm of the counts (normalized using the DESeq2 package in the bioconductor infrastructure) was computed. For statistical assessment of differences between the epochs, the Mann–Whitney *U* test was used; *, *p* < .05; **, *p* < .01. All figures were created using Rstudio 1.1.419 and Ubuntu16.04 as operating system. **(C):** Immunohistochemical expression analysis with HMGCR in retinal organoids at day 90. **(C′):** Inset: high magnification shows wide spread expression from basal to apical layer. **(D):** Expression of HMGCR in fetal retina at 6 PCW; **(E)** 10 PCW; **(F)** 14 PCW; **(G)** 18 PCW in INBZ, ONBZ, and GCL. **(H):** Expression of HMGCR in adult retina in the IS, ONL, INL, and GCL. **(I):** Immunohistochemical analysis for HMGCS1 in retinal organoid at day 90. **(I′):** Inset: high magnification shows widespread expression from the basal to the apical layer. **(J):** Expression of the HMGCS1 in the fetal retina of 6 PCW; **(K)** 10 PCW; **(L)** 14 PCW; and **(M)** 18 PCW, in the INBZ, ONBZ, and GCL. **(N):** Adult human retina shows HMGCS1 immunoreactivity in GCL, IPL, OPL, and IS. Scale bars (C, C′, D, E, F, G, H, I, I′, J, K, L, M, and N): 50 μm. Abbreviations: GFP, green fluorescent protein; ONBZ, outer neuroblastic zone; INBZ, inner neuroblastic zone; PCW, postconception weeks; IS, inner segment; RPE, retinal pigment epithelium; ONL, outer nuclear layer; OPL, outer plexiform layer; INL inner nuclear layer; IPL, inner plexiform layer and GCL, ganglion cell layer. Note: GFP in retinal organoids represents endogenous GFP expression.

Published studies indicate that the retina contains all the genes necessary for independent local cholesterol biosynthesis; however, the rate of cholesterol synthesis is low and extra retinal cholesterol is secured from systemic sources and recycling within the retina 27, 28, 29 and RPE. Inborn errors in cholesterol metabolism (such as abetalipoproteinemia and familial hypobetalipoproteinemia) or exposure to inhibitors of enzymes in the cholesterol can lead to progressive photoreceptor degeneration and apoptosis. Photoreceptors are highly enriched for lipids and different compartments such as the outer and inner segments and their synaptic terminals differ greatly in their lipid content. Within the outer segments (OS), lipids are important for efficient phototransduction as well as generating the second messengers which are involved in signal transduction 29. Notwithstanding, biochemical and molecular studies in human adult retina have demonstrated that cholesterol biosynthesis, catabolism and regulation in the photoreceptor OS are weak and cholesterol content is the lowest of all retinal layers 30, suggesting that cholesterol is likely to be transported into the OS from the inner segments or other retinal cells. Immunohistochemical studies of key components involved in cholesterol homeostasis including HMGCR and LDLR (whose expression is upregulated in cluster 2) have shown widespread expression patterns including the RPE, outer and INL, ganglion cell and nerve fiber layer as well as inner and outer plexiform layers (OPLs) and inner segments and low to absent expression in the OS 30. To gain more insight into the expression of key cholesterol genes enriched in cluster 2, we took advantage of a recent RNA‐seq study performed by our group in human developing retina from 4.6 to 18 postconception week (PCW) 31 to investigate the expression 2 of the differentially expressed genes involved in cholesterol biosynthesis, *HMGCR* and *HMGCS1* (Fig. 5A, 5B), which indicated their peak expression at 7.7–10 PCW, a developmental window during which RGC and cone photoreceptor emergence is observed 31. To investigate the possible cellular localization of HMGCR and HMGCS1, we performed immunocytochemical analysis for two of the differentially expressed genes involved in cholesterol biosynthesis, in day 90 retinal organoids, human fetal retina, and adult retina. In the retinal organoids both proteins were expressed in the plasma membrane of CRX^+^ cells (Fig. 5C, 5C′, 5I, 5I′). Expression in the basal layer of the organoids was also observed and in the case of HMGCS1, the basal expression was much higher than the expression observed in the apical layer of the organoids (Fig. 5I, 5I′) and this was not always restricted to the plasma membrane. In the fetal retina at 6 PCW, both markers were expressed in the inner neuroblastic zone (INBZ) and the outer neuroblastic zone (ONBZ; Fig. 5D, 5J). Both proteins were observed at 10 PCW in all layers with greater immunoreactivity in the INBZ (Fig. 5E, 5K). The intensity of immunostaining was the highest at this developmental stage, corroborating the RNA‐Seq data (Fig. 5A, 5B). Similarly to retinal organoids, expression of both proteins was observed in the plasma membrane of photoreceptor precursors in the ONBZ (Fig. 5E, 5K). From 14 PCW the HMGCR expression was detected in the cell's nucleus in ONBZ, INBZ, and ganglion cell layer (GCL; Fig. 5F), whereas expression of HMGCS1 was observed in the processes of RGCs and INBZ (Fig. 5L). Similarly, expression was found, for both markers, at 18 PCW with immunostaining for HMGCR observed in the photoreceptor precursors, INL and RGCs (Fig. 5G) and for HMGCS1 in the processes of RGCs, INL and RPE (Fig. 5M). In the adult retina, immunoreactivity for HMGCR was detected in the plasma membrane and nuclei of cells throughout the retina (Fig. 5H). In accordance with published data 29, HMGCR expression was observed in inner but not OS of photoreceptors in human adult retina (Fig. 5H) whereas HMGCS1 expression was found in the processes of RGCs and INL (Fig. 5N).

The literature precedence suggest that lipids (for example docosahexaenoic acid) promote photoreceptor differentiation of CRX‐expressing cells both in vitro and in vivo such that CRX expression sets a permissive stage that is essential for photoreceptor differentiation, but which needs other environmental signals (for example lipids) to accomplish further differentiation 30, 31. In light of such results, we are inclined to propose that the higher cholesterol biosynthesis genes in cluster 2 together with lower expression of “photoreceptor/cone markers” may be attributed to a photoreceptor precursor‐like state which is upregulating the cholesterol and lipid biosynthesis as a necessary step for further photoreceptor differentiation. This hypothesis is further corroborated by the significant upregulation of genes involved in mitochondrial biogenesis and/or cristae formation shown in Figure 4C, 4D. The photoreceptor inner segments are rich in mitochondria and changes in mitochondria mass and metabolism are associated with inherited and age‐related retinal dystrophies 32. Photoreceptor differentiation is also associated with exit from cell cycle 33, 34, thus upregulation of genes involved in mitosis and cytokinesis in cluster 2 is highly suggestive of an immature photoreceptor precursor phenotype.

In summary, our single cell RNA‐Seq data of hESC‐derived‐CRX^+^ cells reveals a dominant cell subpopulation with a transcriptional profile consistent with an early cone photoreceptor state and a smaller photoreceptor precursor like cell cluster with lower *CRX* expression which is characterized by higher expression of cholesterol/lipid, mitosis, and mitochondrial biogenesis genes.

### Transplantation of CRX^+^‐GFP hESC‐Derived Photoreceptor Precursors into Adult *Pde6brd1* (C3H) Mice

Robust integration of human photoreceptor precursors in diseased mammalian retina is essential for restoring the visual function and optimizing transplantation therapy; hence, we sought to assess the transplantation capacity of CRX^+^ hESC‐derived photoreceptor precursors into a model of end stage Retinitis Pigmentosa, *Pde6brd1‐*C3H mice. In this mouse model, retinal degeneration is caused by a null mutation in the rod photoreceptor cyclic GMP (cGMP) phosphodiesterase β subunit (*Pde6‐β*) gene 35, 36. This mutation leads to an accumulation of cGMP in the rods and results in photoreceptor cell death 37. The photoreceptor degeneration starts by postnatal day 8 (P8). Ninety‐seven percentage of rod photoreceptors are lost by P17, followed by cone photoreceptor apoptosis around P30 35, 38, resulting in the loss of a functional outer nuclear layer (ONL) by 6–10 weeks of age 39, 40, 41. This fast mode of photoreceptor degeneration provides an optimal model for testing donor photoreceptor engraftment in the absence of material transfer between host and donor photoreceptors which has been shown to account for the majority of donor‐reporter‐labeled cells in the host in nondegenerative animal models 42, 43, 44.

To confirm this fast degeneration, we compared the wild‐type (WT) and *Pde6brd1 (C3H)* retina at 10 postnatal weeks. Immunostaining with pan photoreceptor and cone bipolar marker, Recoverin, confirmed the loss of the ONL in *Pde6brd1* retinae (Supporting Information Fig. S4A, S4B). As expected, no endogenous GFP signal was obtained from the *Pde6brd1* retinae (Supporting Information Fig. S4B). We did not observe any changes in the localization of the rod bipolar cells between the WT and degenerative *Pde6brd1* retinae (Supporting Information Fig. S4C, S4D). A few Recoverin positive cells were observed in the INL of *Pde6brd1* retina; however, these cells did not show immunoreactivity to PKC‐α antibody (Supporting Information Fig. S4E, S4F), which suggests that the remaining Recoverin positive cells are most likely cone bipolar cells. Furthermore, evidence of the photoreceptor loss is the absence of the rhodopsin, opsin blue, and opsin red/green expression in the *Pde6brd1*, compared with the WT which display strong expression of these rod and cone markers in the photoreceptor OS (Supporting Information Fig. S4G, S4H, S4I, S4J, S4K, S4L). The null mutation in *Pde6β* gene in *Pde6brd1* prevents the expression of the functional *PDE6‐β* protein subunit which is normally localized in the photoreceptors OS of WT mice (Supporting Information Fig. S4M, S4N). Synaptophysin is a critical factor for the synaptic vesicle recycling, expressed in the OPL and inner plexiform layer (IPL) of WT mice, which is only observed in the IPL of *Pde6brd1* retinae, most likely due to the absence of the ONL (Supporting Information Fig. S4O, S4P). As expected, there was no immunoreactivity for the human mitochondria in both WT and *Pde6brd1* retinae (Supporting Information Fig. S4Q, S4R). In addition, no changes were observed in the in the GCL at this stage of the degenerative process (Supporting Information Fig. S4S, S4T).

**Figure 6:**
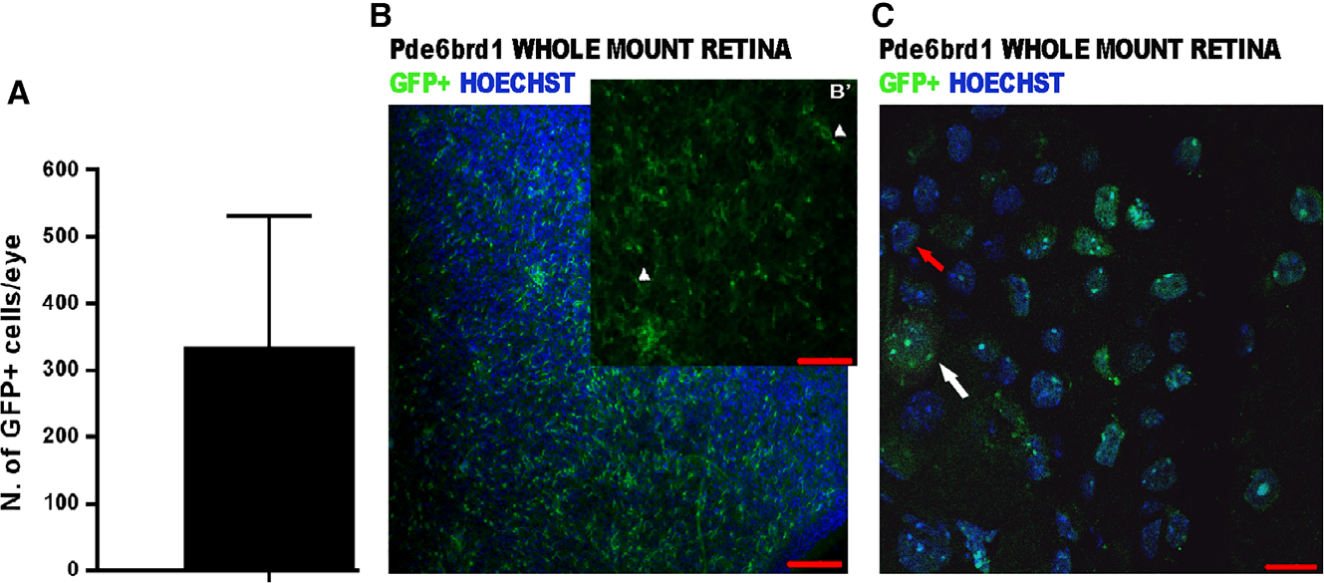
Whole mount analysis of *Pde6brd1* mice subretinally transplanted with hESC‐derived CRX^+^ cells. **(A):** Schematic chart showing the number of GFP^+^ cells per retina. Data is presented as mean ± SD; *n* = 10 mice. **(B, B′):** Whole mount view of GFP^+^ cells found in the retina of *Pde6brd1* mice transplanted with CRX^+^ cells; inset: Individual channel image showing the GFP^+^ cells with the nuclei and processes in green (white arrowheads). **(C):** High magnification image showing the GFP^+^ cells with the green nuclei (white arrow) compared with the nuclei of mouse cells (red arrow). Scale bars: 100 μm (B), 50 μm (B′), and 10 μm (C). Abbreviation: GFP, green fluorescent protein. Note: GFP in (B), (B′) and (C) represents endogenous GFP expression.

At day 90 of differentiation, retinal organoids were collected, dissociated and CRX‐GFP^+^ and CRX‐GFP^−^ cells were isolated by fluorescence‐activated cell sorting as described in Collin et al. 17 and transplanted into the subretinal space of *Pde6brd1* mice. To prevent immune‐rejection of human cells, immunosuppression with cyclosporine was administrated to the host mice through daily subcutaneous injections starting one day prior to the transplantation and continuing throughout the experiment. Three groups of 10 mice each were transplanted with CRX‐GFP^+^, CRX‐GFP^−^ (150,000 cells per eye) or vehicle solution (HBSS), respectively, in both eyes. Three weeks after grafting, mice were sacrificed and host retinae were investigated for human cell engraftment. GFP^+^ cells were found next to the host murine INL in retina (330 ± 196 cells per retina; Fig. 6A) of mice transplanted with CRX‐GFP^+^ cells. No GFP positive cells were found in *Pde6brd1* mice transplanted with CRX‐GFP^−^ cells (data not shown). The GFP^+^ cells were mainly localized around the injection site as shown in the retinal whole mounts analysis (Fig. 6B, 6B′, 6C).

**Figure 7:**
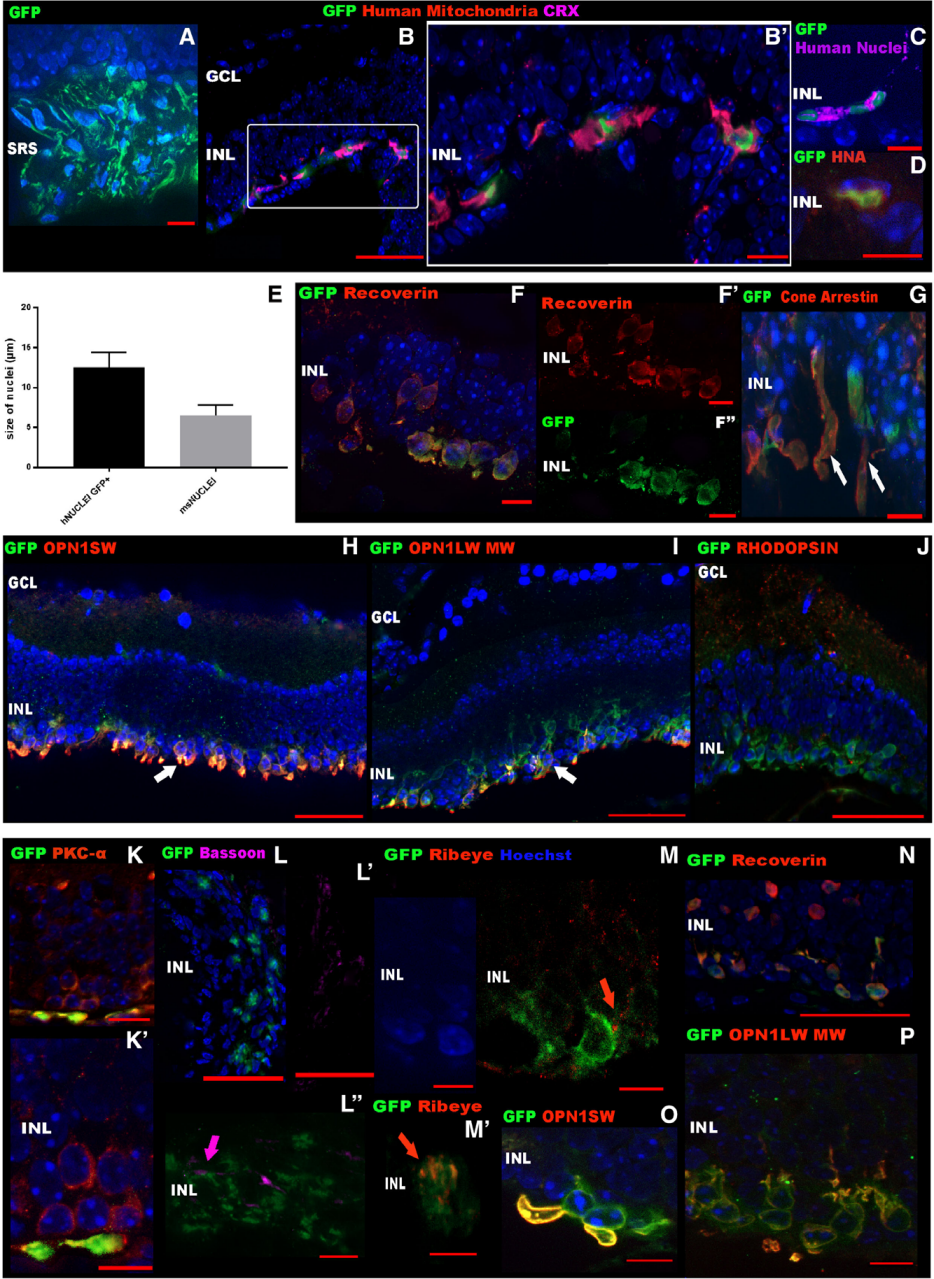
Subretinal transplantation of hESC‐derived CRX^+^ cells into *Pde6brd1* adult mice. **(A):** Low magnification of transplanted retina showing in green the cell mass in the subretinal space (SRS). **(B, B′):** Grafted cells colocalized with the human mitochondria and coexpressed CRX; insets: high magnification images show the immunoreactivity for human mitochondria and CRX in grafted cells. **(C, D):** Expression of human nuclei and human nuclear antigen, respectively. **(E):** Schematic chart showing the difference in size of GFP^+^ and mouse nuclei. Data is presented as mean ± SD. (**F, F′, F″**)**:** Incorporation of GFP^+^ cells and costaining with Recoverin; insets: individual channel images of incorporated cells. **(G):** Transplanted cells coexpress the pan cone marker, cone Arrestin 3 and show typical inner and outer segment expression (white arrows), indicating that human cells are able to acquire a cone photoreceptor phenotype. **(H, I):** Some of the CRX‐GFP^+^ cells coexpress opsin blue (OPN1SW) and opsin red/green (OPN1LW/MW) in vivo. **(J):** No costaining with rhodopsin was observed in the transplanted CRX‐GFP^+^ cells. **(K, K′):** PKC‐α + host bipolar cell were found in close proximity to the GFP^+^ transplanted cells. (K′): Inset: high magnification image showing the CRX‐GFP^+^ in close apposition to the bipolar cells. (**L, L′, L″**)**:** Expression of bassoon (purple), a ribbon synaptic marker, was present between the host INL and the human GFP^+^ cells; insets: (L′) single channel image for bassoon; (L″) higher magnification image showing CRX‐GFP^+^ and the localization of the bassoon (purple arrow). **(M, M′):** Expression of presynaptic protein Ribeye (red) between the CRX‐GFP^+^ cells and the host cells; insets: high magnification images of CRX‐GFP^+^ cells extending neurites which show punctate Ribeye^+^ ribbon synapses (red arrows). **(N):** Incorporation of CRX‐GFP^+^ cells obtained from day 120 organoids and costaining with Recoverin. **(O, P):** Some of the CRX‐GFP^+^ cells obtained from day 120 organoids coexpress opsin blue (OPN1SW) and opsin red/green (OPN1LW MW) in vivo. Scale bars: 50 μm (B, H I, J, K L, L′, and K) and 10 μm (A, B′, C D, F, F′, F″, G, K′, L″, M, M′, O, and P). Abbreviations: GFP, green fluorescent protein; CRX, cone‐rod homeobox; HNA, human nuclear antigen; SRS, subretinal space; INL, inner nuclear layer; GCL, ganglion cell layer. Note: GFP in (A), (B), (B′), (C), (D), (F), (F′), (F″), (G), (H), (I), (J), (K), (K′), (L), (L′), (L″), (M), (M′), (N), (O), and (P) represents endogenous GFP expression.

We also assessed the outcome of the transplantation of CRX‐GFP^+^ cells, in cross‐sections, which showed the presence of the cell mass in close proximity to host retina and donor cells next to the host INL (Fig. 7A). The human origin of these cells was confirmed in retinal cryosections by costaining of human mitochondria, CRX (Fig. 7B, 7B′), human nuclei (Fig. 7C), and human nuclear antigen (HNA; Fig. 7D) and measuring the size of the human GFP^+^ nuclei, in comparison to the mouse nuclei. The mean diameter of the GFP^+^ nuclei was 12.5 μm (±1.87, means ± SD, *n* = 45 nuclei), compared with 6.5 μm (±1.29, means ± SD, *n* = 42 nuclei) for mouse nuclei (Fig. 7E).

The GFP^+^ human cells coexpressed the pan‐photoreceptor marker Recoverin (Fig. 7F, 7F′, 7F″) and began to form a distinct layer in direct contact with the secondary order neurons of the *Pde6brd1* mice also shown by Recoverin expression, representing the host cone bipolar cells. Although the expression of the pan‐cone marker, Arrestin 3, was not found in the CRX‐GFP^+^ cells prior to transplantation (data not shown), strong expression was found in the 33% of the CRX‐GFP^+^ cells in the putative ONL, indicating photoreceptor maturation upon integration into the host retina (Fig. 7G). To confirm that the CRX‐GFP^+^ cells differentiate into cone photoreceptors in vivo, we assessed the presence of the cone opsins: blue (OPN1SW) and red/green (OPN1LW/MW). The cone markers were expressed in the CRX‐GFP^+^ cells and were located in the putative ONL (Fig. 7H, 7I). No rhodopsin immunostaining was observed in the transplanted CRX‐GFP^+^ cells (Fig. 7J), again confirming the cone phenotype of the CRX‐GFP^+^ cells. To transmit a visual signal, the transplanted cells would need to connect with the inner retina of the host, thus to assess donor–host connections we used IHC with a number of synaptic markers. The afferent terminal of host PKC‐α bipolar cells, which is normally in contact with photoreceptors in the INL, was found in the host INL cells at the sites of contact with the donor cells (Fig. 7K, 7K′), indicating a potential mechanism of donor–host interaction. To further explore the anatomy of the synapses, we also verified the expression of Bassoon, an essential component of the ribbon synapse (Fig. 7L, 7L′, 7L″). This analysis showed that ribbon synapses were forming between the CRX‐GFP^+^ cells and host bipolar cells. These data were further corroborated by the immunoreactivity of the presynaptic protein Ribeye, the main protein component of synaptic ribbons 43, that was found in close apposition with the CRX‐GFP^+^ cells and the host INL (Fig. 7M, 7M′) 45. In addition, we transplanted a small group of *Pde6brd1* mice with CRX‐GFP^+^ cells at day 120 of differentiation. As expected, the GFP^+^ human cells coexpressed Recoverin (Fig. 7N), and the opsins: OPN1SW (Fig. 7O) and OPN1LW/MW (Fig. 7P). Together, these data suggest that graft‐host integration occurs within the 3 weeks post‐transplantation, and that hESC‐derived‐CRX^+^ photoreceptor precursors are a promising resource for cone photoreceptor cell replacement in a mammalian model of retinal degeneration.

## Discussion

The application of single cell RNA‐Seq has enabled major advances and better understanding of embryogenesis and stem cell differentiation, immunity, neurobiology, organ development, and tumorigenesis 46, 47, 48, 49, 50. This approach has facilitated the study of complex molecular heterogeneity within tumor, immune, and stem cell compartments as well as enabled identification of new cell types and characteristic markers 51. Retina is particularly suitable for this molecular approach being composed of multiple cell types including photoreceptors, interneurons, ganglion cells, and Müller Glia. A recent single cell genome‐wide expression profiling of mouse retina revealed the presence of 39 cell clusters and novel candidate cell subtypes 52. Similar approaches are being applied to developing human organs 53 and organoids generated from pluripotent stem cells with the hope of better understanding the complexity and emergence of various cellular subtypes during development 18, 54. Deciphering this, wealth of data can often be difficult, as canonical markers to identify cells and precursors at earliest developmental stages are lacking. Often these precursors can present intermediate transient states at low frequency, which necessitates enrichment for adequate analysis. Thus, definition of transcriptomes for retinal cell precursors enriched through well‐defined cell surface markers or fluorescent reporters can be of immense use for generating gene expression trajectories as they emerge during development or differentiation of hESC and hiPSC to retinal organoids.

In this study, we set out to investigate the cellular heterogeneity of CRX expressing cells during differentiation of a hESC reporter line based approach 24. CRX is expressed in photoreceptors and the pinealocytes of the pineal gland 55 and its expression is essential for ensuring high expression of photoreceptor and pineal specific genes, formation of photoreceptor OS and circadian entrainment 56. CRX^+^ cells purified from mouse embryonic retina are found into the ONL of recipient retina where they express cone markers 20; however, postnatal CRX^+^ cells generate a much higher number of rods, suggesting that CRX^+^ cells may be suitable for replacement of lost cones or rods depending on their stage of developmental maturation. Since markers of cone and rod photoreceptors are well delineated in both mouse and human, we anticipated that single cell RNA‐Seq of CRX^+^ cells isolated from hESC‐derived retinal organoids would not only reveal the heterogeneity within this population, but also define the developmental maturation of these cells and thus inform the transplantation outcome. Our single cell RNA‐seq data analysis revealed a dominant cell cluster within the CRX^+^ cells at day 90 of differentiation, which contained 72% of cells and showed a high expression of genes expressed in early cone photoreceptors. A smaller cluster containing 28% of the cells displayed lower expression of photoreceptor markers and a significantly higher expression of genes involved in cholesterol/lipid biosynthesis, mitochondrial biogenesis, and cell cycle genes which we associated with an “earlier precursor”‐like state. At 3 weeks post‐transplantation into the subretinal space of 8 to 10‐week‐old *Pde6brd1* mice, human GFP^+^ cells expressing the pan‐photoreceptor marker Recoverin were found in a distinct outer nuclear like layer in direct contact with the host secondary order neurons. Some of the transplanted human CRX^+^ cells displayed the expression of pan cone photoreceptor marker, Arrestin 3, and cone opsins, which indicates further differentiation to more mature cones upon transplantation and corroborates recent data obtained with mouse ESC and retinal‐derived CRX‐GFP^+^ photoreceptor precursors 11. Since no cones were found in the host retina, it is impossible for the CRX‐GFP^+^ to have acquired the cone fate through cellular transfer.

The question arises as to why these CRX^+^ cells mature into cones upon transplantation into this animal model of retinal degeneration. The most likely explanation is that CRX‐GFP^+^ cells were transplanted when committed to an early cone fate as shown by the single cell RNA‐Seq analysis. Since cones emerge before rods during retinogenesis, it can be argued that CRX^+^ photoreceptor precursors first express cone markers and later during differentiation acquire the expression of rod precursor markers which may facilitate their differentiation into mature rods upon transplantation. Single cell transcriptomic analysis of CRX^+^ enriched from early (day 70) and late stage (day 218) retinal organoids did not reveal this to be the case 22: at both stages, CRX expression was associated and most similar with cone markers which may indicate a transcriptional driven propensity of CRX^+^ cells to differentiate into cone photoreceptors in retinal organoids up to day 218 of differentiation at least. Mouse studies have, however, indicated that postnatal CRX^+^ do give rise to rods upon transplantation, therefore it remains to be determined if CRX^+^ cells enriched from much later stages of retinal organoid differentiation (after day 270) express rod marker genes. This is corroborated by single cell analysis of adult retina 22, which shows predominant expression of rod marker genes in the CRX^+^ photoreceptors, suggesting a change in transcriptomic profile as these cells mature during development.

In addition to developmental maturation, it is of interest to investigate the role of host retina. In this study, we used a model of fast retinal degeneration to ensure lack of host photoreceptors and minimize the likelihood of material transfer between host and donor photoreceptors, thus enhancing the possibility of hESC‐derived photoreceptor precursor engraftment 11, 42, 43. We performed immunocytochemistry using two different antibodies to HNAs and in both cases we observed colocalization with the CRX immunostaining and endogenous GPP expression, thus demonstrating that these cells were not endogenous mouse photoreceptors and did not arise through material transfer. We did not observe any polyploid nuclei, thus excluding cell fusion as potential mechanism for the presence of CRX‐GFP^+^ cells next to the host INL. In a recently published article, Waldron et al. (2018) show that the retinal environment of *Nrl* ^*−/−*^ and *Prph2* ^*rd2/rd2*^ models supports both donor cone‐derived photoreceptor integration alongside material transfer, which the authors associated with a cone rich retinal environment and a disrupted OLM due to injection trauma 11. The *Pde6brd1* mouse model is different to the two models described above in that it degenerates extremely fast and thus is unlikely to host cone or rod cells which would enable material transfer with the transplanted human cells. Recent papers have also shown that material transfer is a developmentally regulated process with cones derived from postnatal stages engaging in material transfer more frequently than immature retinal cells 11, 57. Our molecular analysis indicates an early cone like transcriptomic profile for CRX^+^ cells at day 90 of differentiation, which could also underline the lack of material transfer in addition to degenerative nature of the *Pde6brd1* mouse model.

## Conclusion

Collectively, our data provide new insights into the transcriptional profile of CRX^+^‐derived pluripotent stem cell photoreceptor precursors and provide evidence of their usefulness as a source of transplantable cone photoreceptors. Future transplants into various animal models of retinal degeneration as well as behavioral, electrophysiological, and functional analyses are required to determine the presence of any light response from these grafts and the feasibility of this approach.

## Author Contributions

J. Collin: experimental design, performed research, data collection and analysis and manuscript writing, approved the final version of the manuscript; D.Z.: experimental design, performed research, data collection and analysis, figure preparation and manuscript writing, approved the final version of the manuscript; R.Q.: data analysis, figure preparation and manuscript writing, approved the final version of the manuscript; T.S.‐F., C.M.: experimental design, approved the final version of the manuscript; R.B.: data analysis, approved the final version of the manuscript; J. Coxhead: experimental design, performed research and data collection, approved the final version of the manuscript; R.H. and D.S.: performed research and data collection, approved the final version of the manuscript; M.A. and E.S.: experimental design and data analysis, approved the final version of the manuscript; L.A.: performed research, data collection, experimental design and data analysis, approved the final version of the manuscript; M.L.: study design, data analysis, figure preparation, manuscript writing and fund raising, approved the final version of the manuscript.

## Disclosure of Potential Conflicts of Interest

The authors indicated no potential conflicts of interest.

## Supporting information


**Figure S1: Stepwise filtering strategy of single cell RNA‐Seq data**. **A** and **B**) Cells which had fewer than 150,000 reads or 2,000 genes were removed from downstream analysis; **C**) Cells with housekeeping genes of less than 20,000 counts were removed from analysis; **D**) Cells with higher than 10% mitochondrial genes were removed from analysis; **E**) Cells with higher than 25% of Ambion spikes were removed from analysis; **F**) Total gene expression before and after data normalization.

**Figure S2: CRX‐GFP** ^**+**^ **cells within cluster 1 and 2 show a similar expression of rod precursor marker NRL. A**) Expression of *NRL* in cluster 1 and 2 shown through a violin plot profile; **B**) Immunostaining of retinal organoids at day 90 showing few GFP^+^ cells co‐stained with NRL (white arrows); **B′**) Inset: high magnification of NRL^+^ and CRX‐GFP^+^ cell. Scale bars, 50 μm (B) and 10 μm (B′). Abbreviations: GFP, green fluorescent protein. *Note: GFP in B and B′ represents endogenous GFP expression*.

**Figure S3: CRX‐GFP** ^**+**^ **cells within cluster 1 show a cone biased transcriptional profile. A**) Expression of *ONECUT1, OLIG2* and *OTX2* cone markers in cluster 1 and 2 shown through violin plot profiles; **B**) Immunostaining of retinal organoids at day 90 showing co‐staining of CRX‐GFP^+^ cells with OTX2; very few CRX‐GFP^+^ cells co‐localize with ONECUT1 (white arrows) and OLIG2 (white arrows). Abbreviations: GFP, green fluorescent protein. Scale bars, 50 μm (B). *Note: GFP in B represents endogenous GFP expression*.

**Figure S4: Characterization of *Pde6brd1* Mouse Model of Retinal Degeneration compared with Wild Type Mouse Retina.** IHC imaged showing the different localization of retinal markers in *Pde6brd1* Mouse Model of Retinal Degeneration and C57 Wild Type Mouse (WT). **A‐B**) Localization of pan‐photoreceptor marker (Recoverin) in WT retina in the OS/IS and ONL (**A**) and in *Pde6brd1* retina (**B**); **C‐D**) Localization of PKC‐α + cells in rod bipolar cells in WT retina (**C**) and *Pde6brd1* retina (**D**); **E‐F**) Co‐immunostaining for Recoverin (red) and PKC‐α (green) in the WT mice (**E**). In the *Pde6brd1* retina (**F**) the remaining Recoverin+ cells co‐stained with the bipolar cell marker in the INL; **G‐H**). Expression of Rhodopsin marker (red) in WT retina (**G**) in the OS and lack of expression in the retinae of *Pde6brd1* mice retina (**H**); **I‐J‐K‐L**) Localization of the opsins blue (red) and red/green (red) in WT retina (**I‐K**) in the photoreceptor OS. Both opsins are completely absent in the *Pde6brd1* retina (**J‐L**); **M‐N**); PDE6‐ β is localized in the OS in WT retina (**M**), but is completely absent in *Pde6brd1* retina (**N**); **O‐P**) Expression of Synaptophysin in the OPL and IPL in WT retina (**O**) and only in the IPL in *Pde6brd1* retina (**P**); **Q‐R**) Reactivity to human mitochondrial antigen is absent in WT and *Pde6brd1* retina; **S‐T**) Localization of RBPMS in the retinal ganglion cells in WT retina (**S**) and *Pde6brd1* retina (**T**); Scale bars 50 μm (A, B, C, D, E, F, G, H, I, J, K, L, M, N, O and P) . Abbreviations: GFP, green fluorescent protein; RPE, retinal pigment epithelium; OS, outer segment; IS, inner segment; ONL, outer nuclear layer; OPL, outer plexiform layer; INL inner nuclear layer; IPL, inner plexiform layer and GCL, ganglion cell layer.

**Table S1:** Summary of antibodies used for immunohistochemical staining.

**Table S2:** List of significantly and differentially expressed genes between clusters 1 and 2.
