# Supplementary figures and images for: CRX Expression in Pluripotent Stem Cell‐Derived Photoreceptors Marks a Transplantable Subpopulation of Early Cones
Source: Stem Cells. 2019 Jan 30;37(5):609–22. doi: 10.1002/stem.2974 (PMC6519156; doi:10.1002/stem.2974)

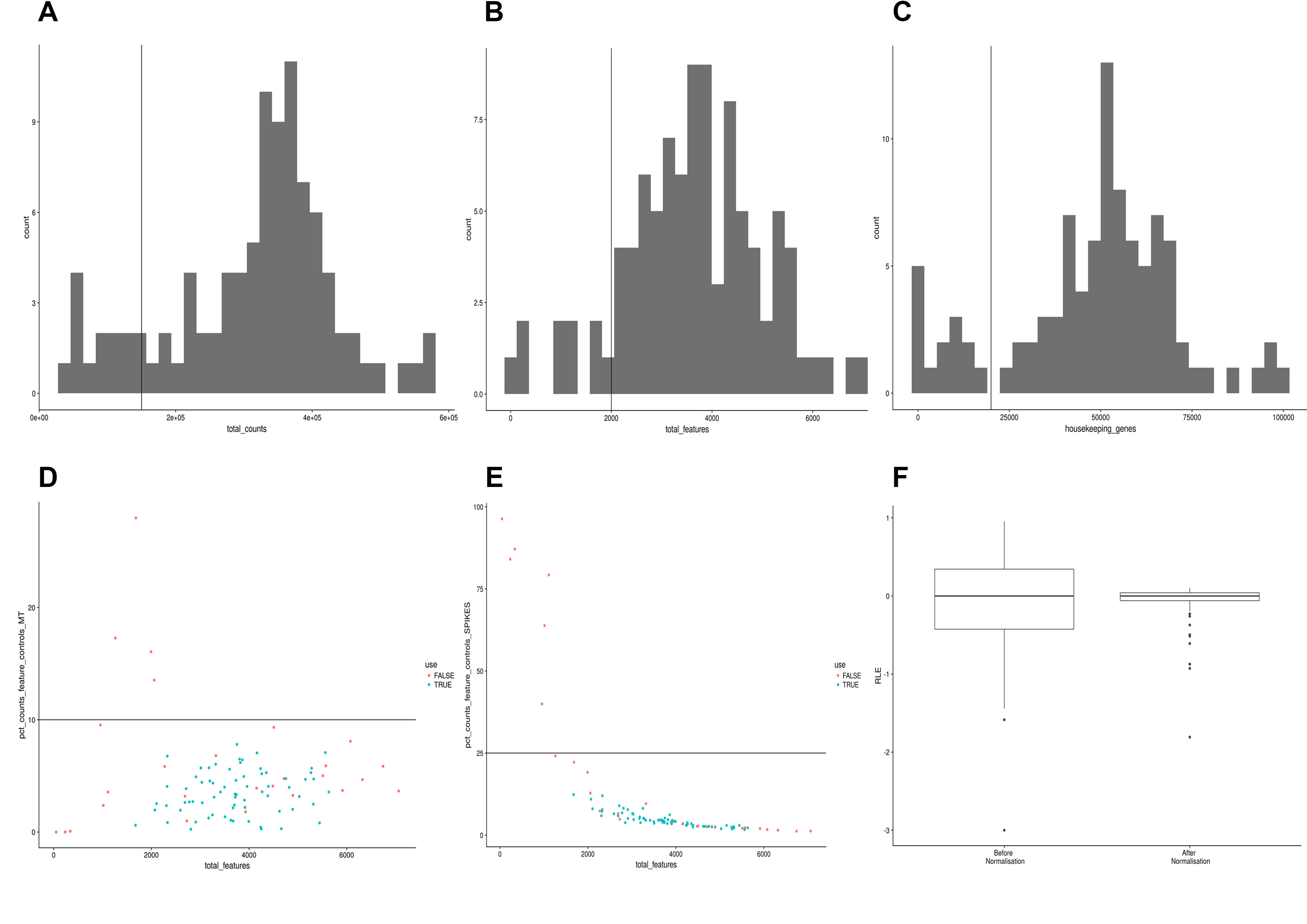

Supplement: Supplementary file 1 — Figure S1: Stepwise filtering strategy of single cell RNA‐Seq data. A and B) Cells which had fewer than 150,000 reads or 2,000 genes were removed from downstream analysis; C) Cells with housekeeping genes of less than 20,000 counts were removed from analysis; D) Cells with higher than 10% mitochondrial genes were removed from analysis; E) Cells with higher than 25% of Ambion spikes were removed from analysis; F) Total gene expression before and after data normalization. [file STEM-37-609-s001.jpg]

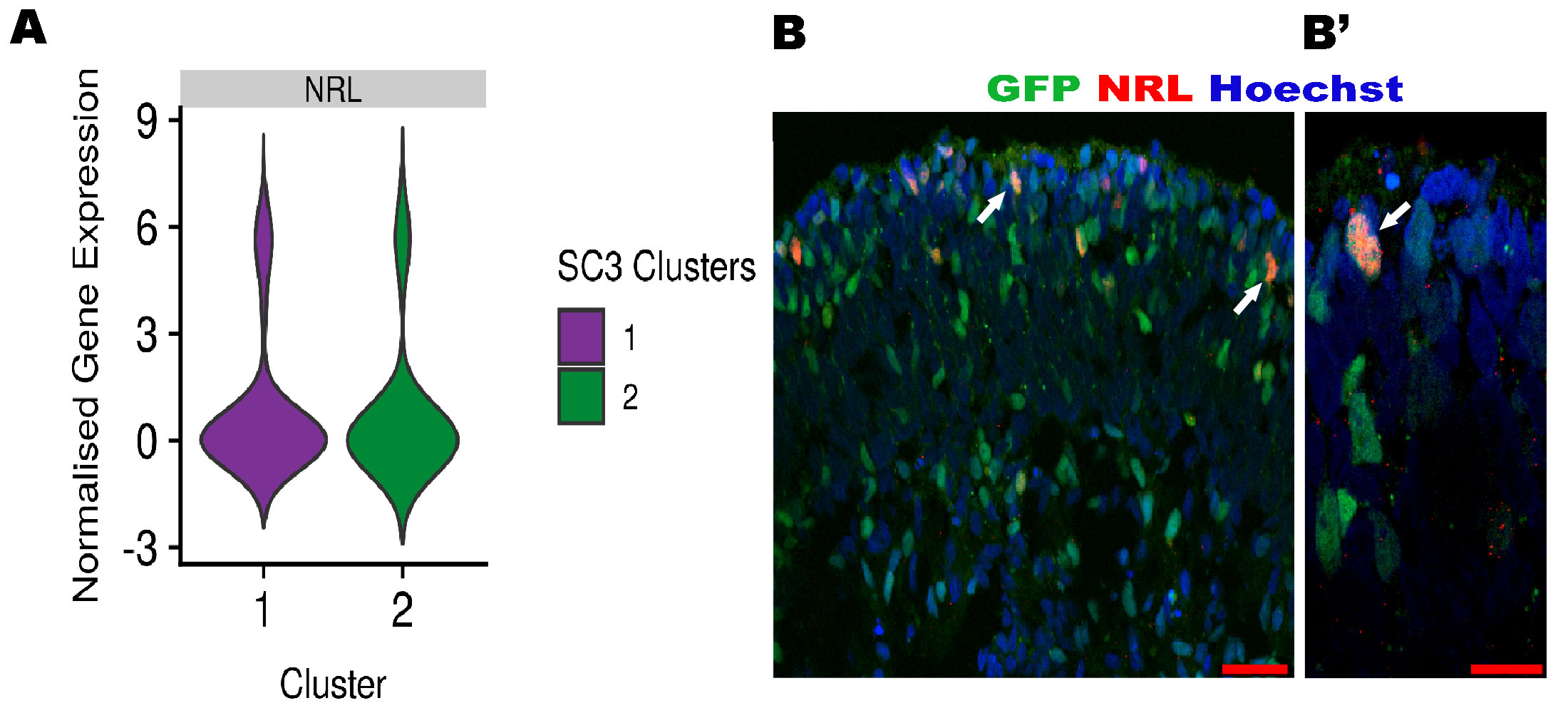

Supplement: Supplementary file 2 — Figure S2: CRX‐GFP + cells within cluster 1 and 2 show a similar expression of rod precursor marker NRL. A) Expression of NRL in cluster 1 and 2 shown through a violin plot profile; B) Immunostaining of retinal organoids at day 90 showing few GFP+ cells co‐stained with NRL (white arrows); B′) Inset: high magnification of NRL+ and CRX‐GFP+ cell. Scale bars, 50 μm (B) and 10 μm (B′). Abbreviations: GFP, green fluorescent protein. Note: GFP in B and B′ represents endogenous GFP expression. [file STEM-37-609-s002.jpg]

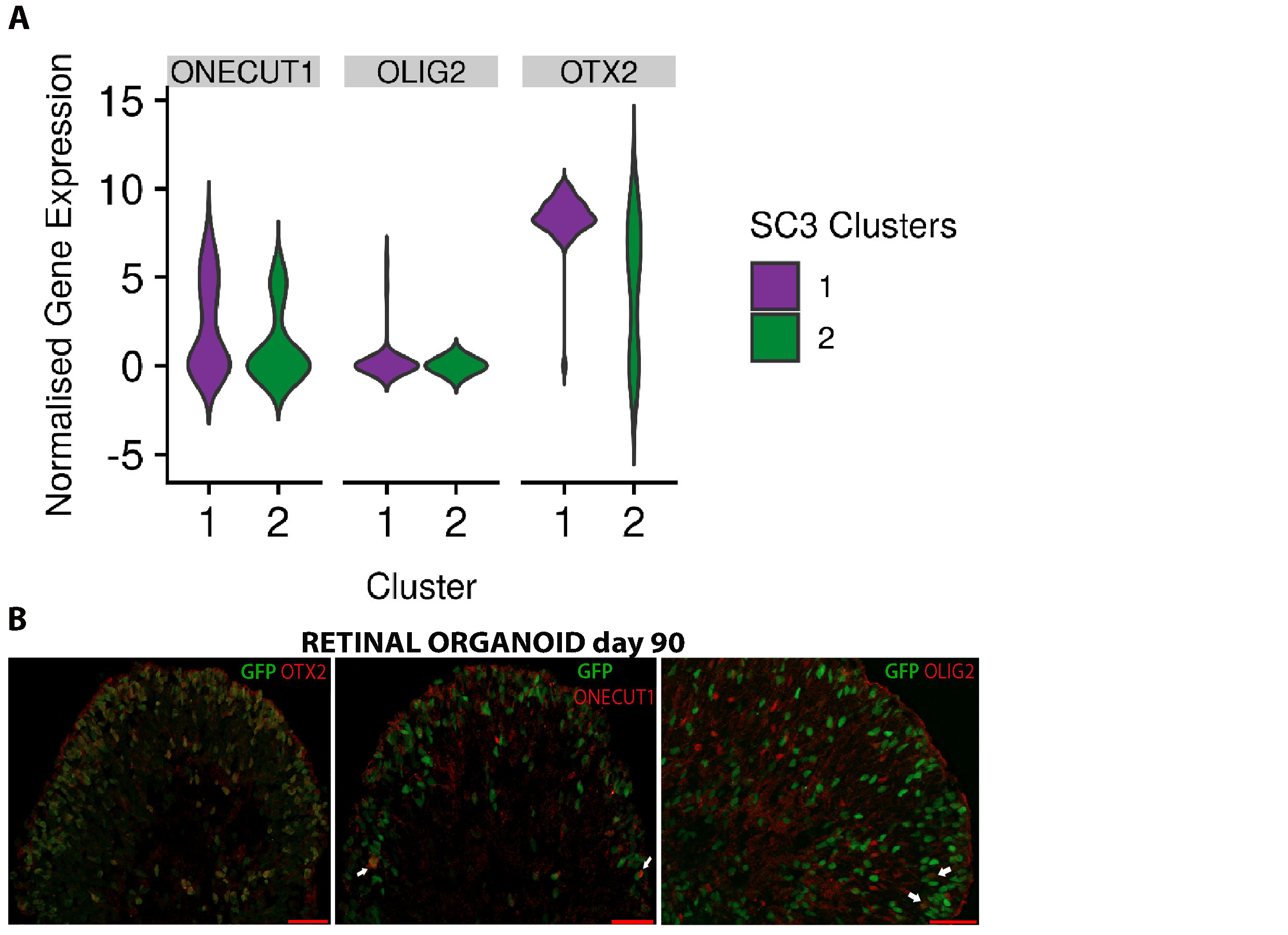

Supplement: Supplementary file 3 — Figure S3: CRX‐GFP + cells within cluster 1 show a cone biased transcriptional profile. A) Expression of ONECUT1, OLIG2 and OTX2 cone markers in cluster 1 and 2 shown through violin plot profiles; B) Immunostaining of retinal organoids at day 90 showing co‐staining of CRX‐GFP+ cells with OTX2; very few CRX‐GFP+ cells co‐localize with ONECUT1 (white arrows) and OLIG2 (white arrows). Abbreviations: GFP, green fluorescent protein. Scale bars, 50 μm (B). Note: GFP in B represents endogenous GFP expression. [file STEM-37-609-s003.jpg]

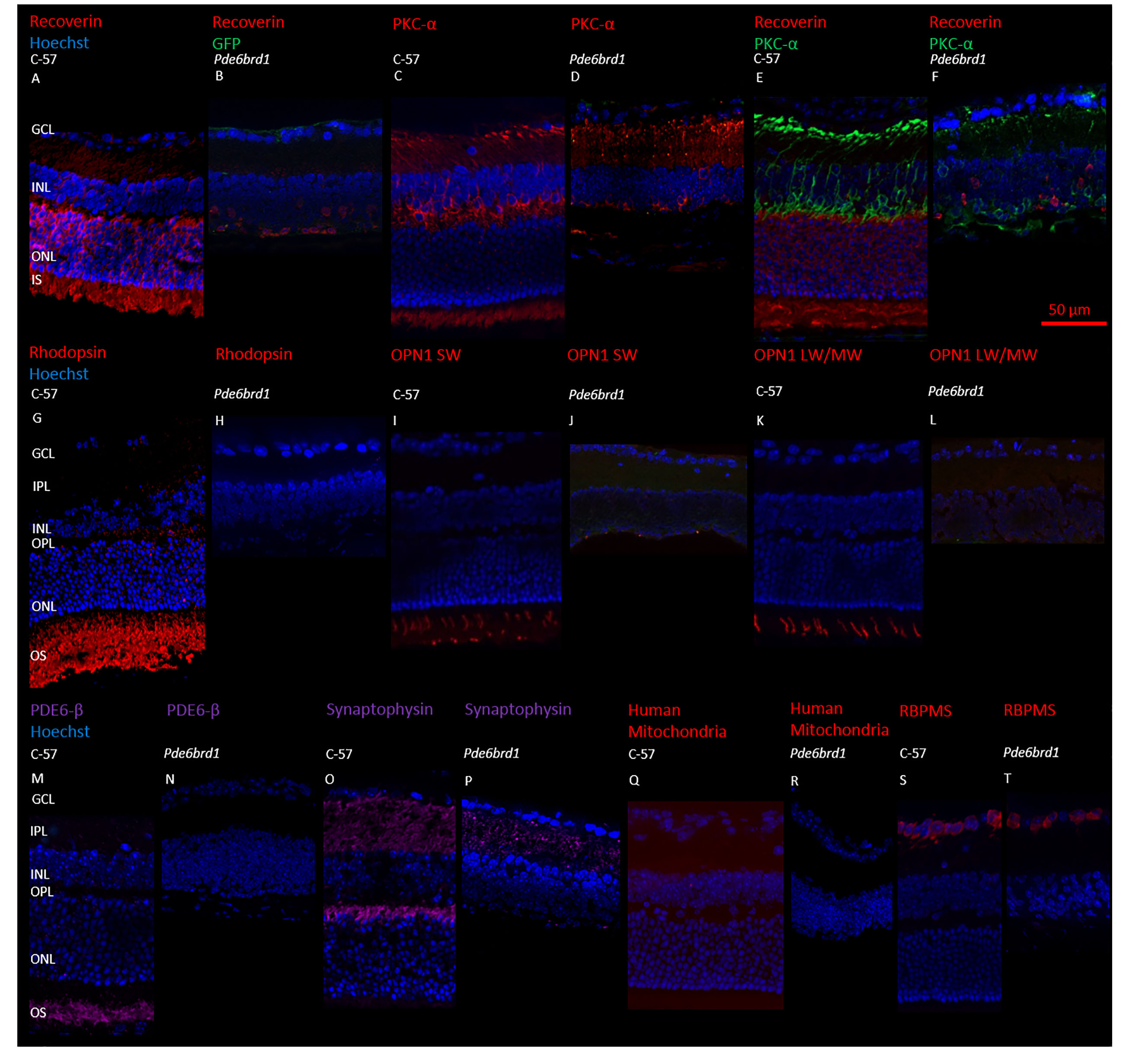

Supplement: Supplementary file 4 — Figure S4: Characterization of Pde6brd1 Mouse Model of Retinal Degeneration compared with Wild Type Mouse Retina. IHC imaged showing the different localization of retinal markers in Pde6brd1 Mouse Model of Retinal Degeneration and C57 Wild Type Mouse (WT). A‐B) Localization of pan‐photoreceptor marker (Recoverin) in WT retina in the OS/IS and ONL (A) and in Pde6brd1 retina (B); C‐D) Localization of PKC‐α + cells in rod bipolar cells in WT retina (C) and Pde6brd1 retina (D); E‐F) Co‐immunostaining for Recoverin (red) and PKC‐α (green) in the WT mice (E). In the Pde6brd1 retina (F) the remaining Recoverin+ cells co‐stained with the bipolar cell marker in the INL; G‐H). Expression of Rhodopsin marker (red) in WT retina (G) in the OS and lack of expression in the retinae of Pde6brd1 mice retina (H); I‐J‐K‐L) Localization of the opsins blue (red) and red/green (red) in WT retina (I‐K) in the photoreceptor OS. Both opsins are completely absent in the Pde6brd1 retina (J‐L); M‐N); PDE6‐ β is localized in the OS in WT retina (M), but is completely absent in Pde6brd1 retina (N); O‐P) Expression of Synaptophysin in the OPL and IPL in WT retina (O) and only in the IPL in Pde6brd1 retina (P); Q‐R) Reactivity to human mitochondrial antigen is absent in WT and Pde6brd1 retina; S‐T) Localization of RBPMS in the retinal ganglion cells in WT retina (S) and Pde6brd1 retina (T); Scale bars 50 μm (A, B, C, D, E, F, G, H, I, J, K, L, M, N, O and P) . Abbreviations: GFP, green fluorescent protein; RPE, retinal pigment epithelium; OS, outer segment; IS, inner segment; ONL, outer nuclear layer; OPL, outer plexiform layer; INL inner nuclear layer; IPL, inner plexiform layer and GCL, ganglion cell layer. [file STEM-37-609-s004.jpg]
