# Supplementary material for: CRX Expression in Pluripotent Stem Cell‐Derived Photoreceptors Marks a Transplantable Subpopulation of Early Cones
Source: Stem Cells. 2019 Jan 30;37(5):609–22. doi: 10.1002/stem.2974 (PMC6519156; doi:10.1002/stem.2974)
Supplement: Supplementary file 5 — Table S1: Summary of antibodies used for immunohistochemical staining. [file STEM-37-609-s005.docx]

**Table S1**

| **Antibody** | **Host** | **Dilution** | **Supplier, Cat. No** |
| --- | --- | --- | --- |
| ANTI-Human Nuclei | Mouse | 1:100 | Millipore, MAB1281 |
| Anti- Human Mitochondria | Rabbit | 1:100 | Millipore, AB3598 |
| Anti- Synaptophysin | Rabbit | 1:200 | abcam, ab32127 |
| Anti-Opsin red/green | Rabbit | 1:200 | abcam, ab5407 |
| Anti-Opsin blue | Rabbit | 1:200 | abcam, ab5405 |
| Anti-PDE6-β | Rabbit | 1:100 | abcam, ab5663 |
| Anti- Human Nuclear Antigen | Mouse | 1:100 | abcam, ab191181 |
| Anti-Arrestin 3 | Rabbit | 1:100 | Novus Biological, NBP2-41249 |
| Anti-Recoverin | Rabbit | 1:1000 | Millipore, AB5585 |
| Anti-Rhodopsin | Rabbit | 1:100 | Abcam, ab59260 |
| Anti-Crx | Rabbit | 1:25 | Santa Cruz, SC-30150 |
| Anti-PKCα | Mouse | 1:200 | BD Transduction laboratories, 610107 |
| Anti-RBPMS | Rabbit | 1:1000 | PhosphoSolutions, 1830-RBPMS |
| Anti-Bassoon | Mouse | 1:100 | StressGen, VAM-PS003 |
| Anti-Ribeye | Mouse | 1:100 | BD Bioscience, 612044 |
| Anti-HMGCR | Rabbit | 1:500 | Abcam, ab214018 |
| Anti-HMGCS1 | Rabbit | 1:100 | Abcam, ab87246 |
| Anti-Otx2 | Rabbit | 1:200 | Abcam, ab114138 |
| Anti-Olig2 | Rabbit | 1:500 | Millipore, AB9610 |
| Anti-HNF-6 | Mouse | 1:200 | Santa Cruz, sc-376167 |
